# Massive *Trentepohlia*-Bloom in a Glacier Valley of Mt. Gongga, China, and a New Variety of *Trentepohlia* (Chlorophyta)

**DOI:** 10.1371/journal.pone.0037725

**Published:** 2012-07-16

**Authors:** Guoxiang Liu, Qi Zhang, Huan Zhu, Zhengyu Hu

**Affiliations:** 1 State Key Laboratory of Freshwater Ecology and Biotechnology, Institute of Hydrobiology, Chinese Academy of Sciences, Wuhan, Hubei, People’s Republic of China; 2 Graduated School, Chinese Academy of Sciences, Beijing, People’s Republic of China; University of New South Wales, Australia

## Abstract

*Trentepohlia* is a genus of subaerial green algae which is widespread in tropical, subtropical, and also temperate regions with humid climates. For many years, small-scale *Trentepohlia* coverage had been found on the rocks of some glacier valleys on the northern slopes of Mt. Gongga, China. However, since 2005, in the Yajiageng river valley, most of the rocks are covered with deep red coloured algal carpets, which now form a spectacular sight and a tourist attraction known as ‘Red-Stone-Valley’. Based on morphology and molecular data, we have named this alga as a new variety: *Trentepohlia jolithus* var. *yajiagengensis* var. nov., it differs from the type variety in that its end cells of the main filament are often rhizoid, unilateral branches. This new variety only grows on the native rock, both global warming and human activity have provided massive areas of suitable substrata: the rocks surfaces of the Yajiageng river valley floodplain were re-exposed because of heavy debris flows in the summer of 2005; plus human activities such as tourism and road-building have also created a lot of exposed rock! In summer, the glaciers of the northern slopes of Mt. Gongga have brought to the valleys wet and foggy air, ideal for *Trentepohlia* growth. The cells of the new variety are rich in secondary carotenoids (astaxanthin?), which helps the algal cells resistance to strong ultraviolet radiation at high altitudes (they are only found on rock surfaces at alt. 1900–3900 m); the cells are also rich in oils, which gives them high resistance to cold dry winters.

## Introduction

*Trentepohlia* Martius 1817 is a common genus of subaerial green algae, and is widespread in regions with humid climates and grows on wood, tree bark, leaves, rock, building walls and several other types of artificial substrata [1], [2]. Generally, it is most abundant and diverse in the tropics [3], [4], however, it is also present in temperate regions [5], [6]. As presently circumscribed, *Trentepohlia* is the largest genus (about 35 species) in the order Trentepohliales which included one family, Trentepohliaceae, and six genera.

*Trentepohlia* has been studied intensively; however, information concerning the biology of the genus is still limited. Shape and size of vegetative cells, presence of hair-like cells (setae), branch pattern, position and morphology of reproductive structures are considered the most important characteristics for identification at species level [6]. However, several morphological characteristics have shown remarkable variation in relation to the environmental conditions [7]. *Trentepohlia jolithus* (Linnaeus) Wallroth 1833 ( =  *Byssus jolithus* Linnaeus 1753) is one of two originally described species (the other species being *Trentepohlia aurea* (Linnaeus) Martius 1833). *Trentepohlia jolithus* grows on dead wood, rock, stone, concrete or cement walls, and other solid substrata [6], [8]. However, it fails to grow on any living plant [8]. *T*. *jolithus* can form deep red patches, and in some years, can grow to cover many square meters of surface. Consequently, they are easy to recognize by the trained eye because of their characteristic appearance that this species forms deep red velvety covering with irregular vertical streaks [6]. This species is extensively distributed in tropical and temperate regions such as New Zealand [8], India [9], Japan [10], Europe [11], and the USA [12].

Mt. Gongga (aka Mt. Konka, 29°20′–30°20′ N, 101°30′–102°15′ E, alt. 7556 m) is the highest peak located on the south-eastern-fringe of the Tibetan Plateau. It is a border mountain, and one of the easternmost glaciated areas in China, in the transitional zone between the dry Tibetan Plateau and the humid Sichuan basin [13]. The great span in altitude (1100–7556 m) has resulted in diverse vertical vegetation zones, with the forest types ranging from subtropical vegetation to alpine cold vegetation [14]. This area also possesses an integrated primary succession sequence from pioneer community to climax community [15]. According to the weather station located at 3000 m, the mean annual precipitation is 1925 mm, most of which (approx. 80%) falls between June and October, and annual potential evaporation averages 264 mm. The mean monthly temperature ranges from −4.5°C in January to 12.7°C in July [16]. The annual evaporation is relatively small (about 300 mm), and the annual average relative humidity is above 90% [17]. On the northern slopes of Mt. Gongga, *Trentepohlia* has been found on the rocks of some glacier valleys, forming conspicuous red cushions. Furthermore, in the whole of the valley of the Yajiageng river, the occurrence of dense *Trentepohlia* blooms has been noted. Most of the rocks are covered with deep red colored algal carpets which extend for many kilometers along the valley. This spectacular sight, known as ‘Red-Stone-Valley’ attracts many tourists every year.

In October 2010, we collected *Trentepohlia* in the Yajiageng valley, Mt. Gongga. The morphological information obtained in this investigation was acquired by specimen examination and culture observation. After morphological comparison, we have described it as a new variety, and then discussed the particular ecological character of this variety; and examined the reason for the massive and extensive growth of *Trentepohlia* in these particular glacier valleys. Based on the nuclear small subunit rRNA gene (SSU rDNA, or 18S rDNA) the molecular phylogenies, including this variety and other taxa of the order Trentepohliales, had been discussed.

## Results

### Taxonomic Description

Trentepohlia jolithus (Linnaeus) Wallroth var. yajiagengensis var. nov.

Var. paries cellulae crassus, exasperatumque, apical cellulas principalis filament interdum rhizoid.

#### Holotype

China. SC-2011-001 (HBI) (29°50′16′′ N, 102°02′35′′ E, alt. 3004 m), from Mt. Gongga, collected by Guo-Xiang Liu on October 24, 2010. It is kept in Freshwater Algal Herbarium (HBI), Institute of Hydrobiology, Chinese Academy of Sciences, Wuhan, Hubei, China.

#### Etymology

The variety epithet *yajiagengensis* refers to the geographical origin (The valley of the Yajiageng river, Mt. Gongga, China).

#### Distribution

**Figure 1:**
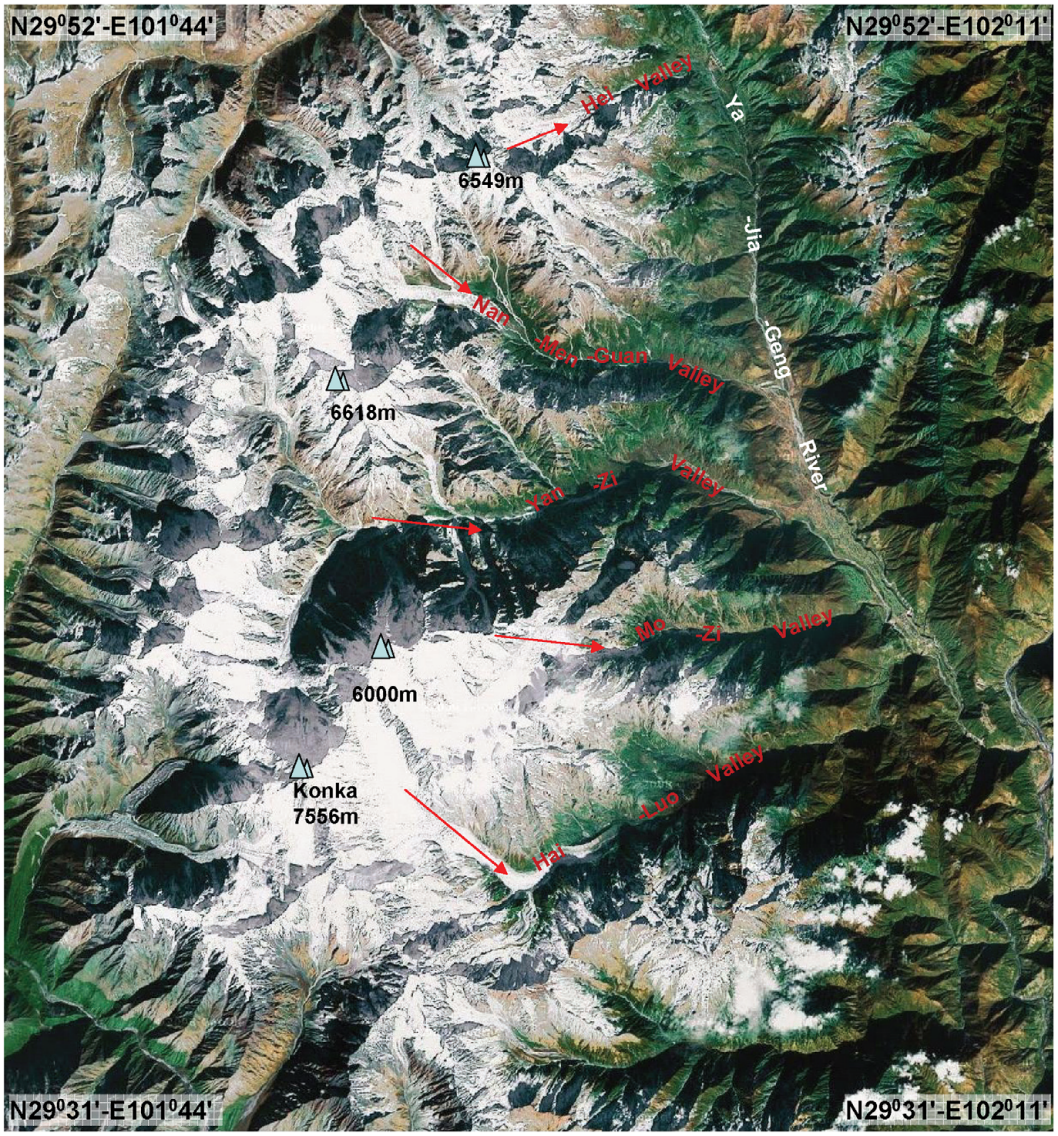
Map of Gongga Mountain with several glacier valleys, where *Trentepohlia*-bloom was found. (taken from Google™ earth).

Several glacier valleys of Mt. Gongga, China (Fig. 1).

**Figure 2:**
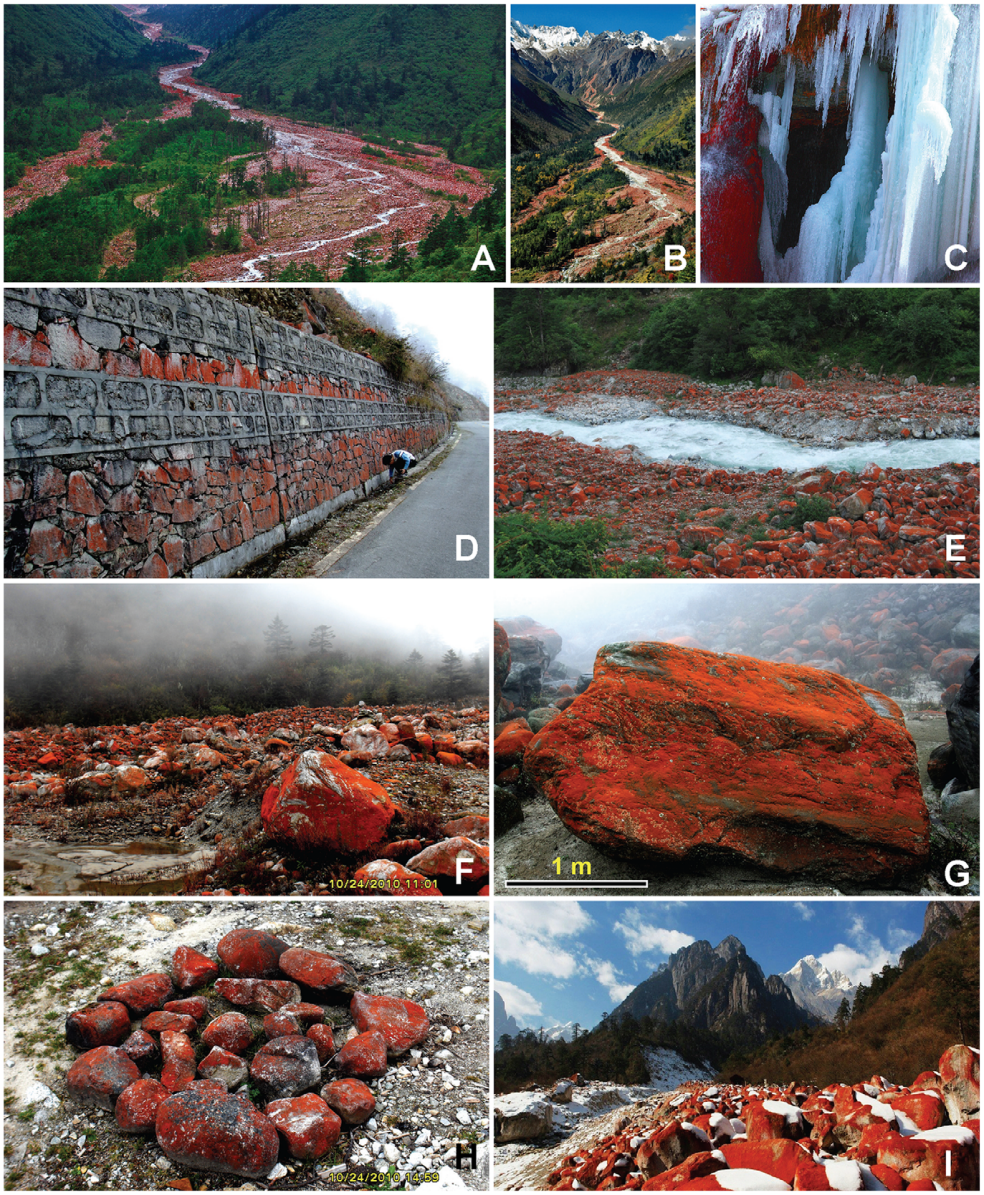
Red-Stone-Valley and the stones covered with *Trentepohlia*-carpets. **2A-2B:** Red-stone Valley and the Yajiageng River; 2**C:** Red *Trentepohlia*-carpet in a cold winter; **2D:** *Trentepohlia* growing on stone walls near the road; **2E:** Red-Stone-Valley and Yajiageng River; **2F-2G:** Red-Stone-Valley in foggy conditions; **2H:** Tibetan Ni-ma stack with *Trentepohlia* growing on it. **2I:** Red-Stone-Valley in winter.

**Figure 3:**
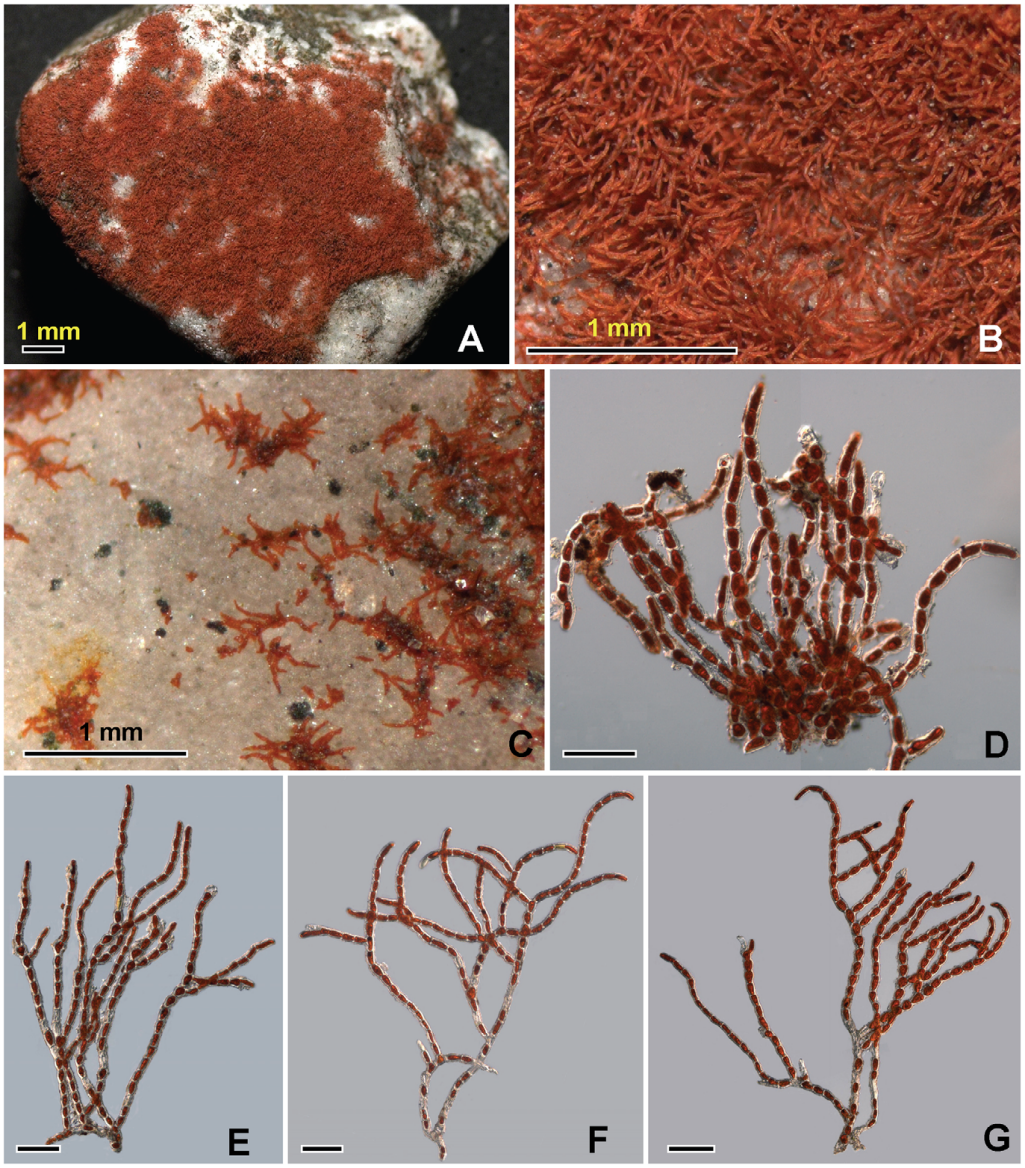
The filamentous of *Trentepohlia jolithus* var. *yajiagengensis* var. nov. 3A: A small piece of gravel covered with *Trentepohlia* under the stereoscope; **3B:** *Trentepohlia*-carpet under the stereoscope; **3C:** Primary filaments of *Trentepohlia* creeping on a stone surface; **3D:** Tangle filaments of *Trentepohlia* with irregular prostrate cells; **3E-3G:** Erect filaments of *Trentepohlia*. All scale bars  = 100 µm.

**Figure 4:**
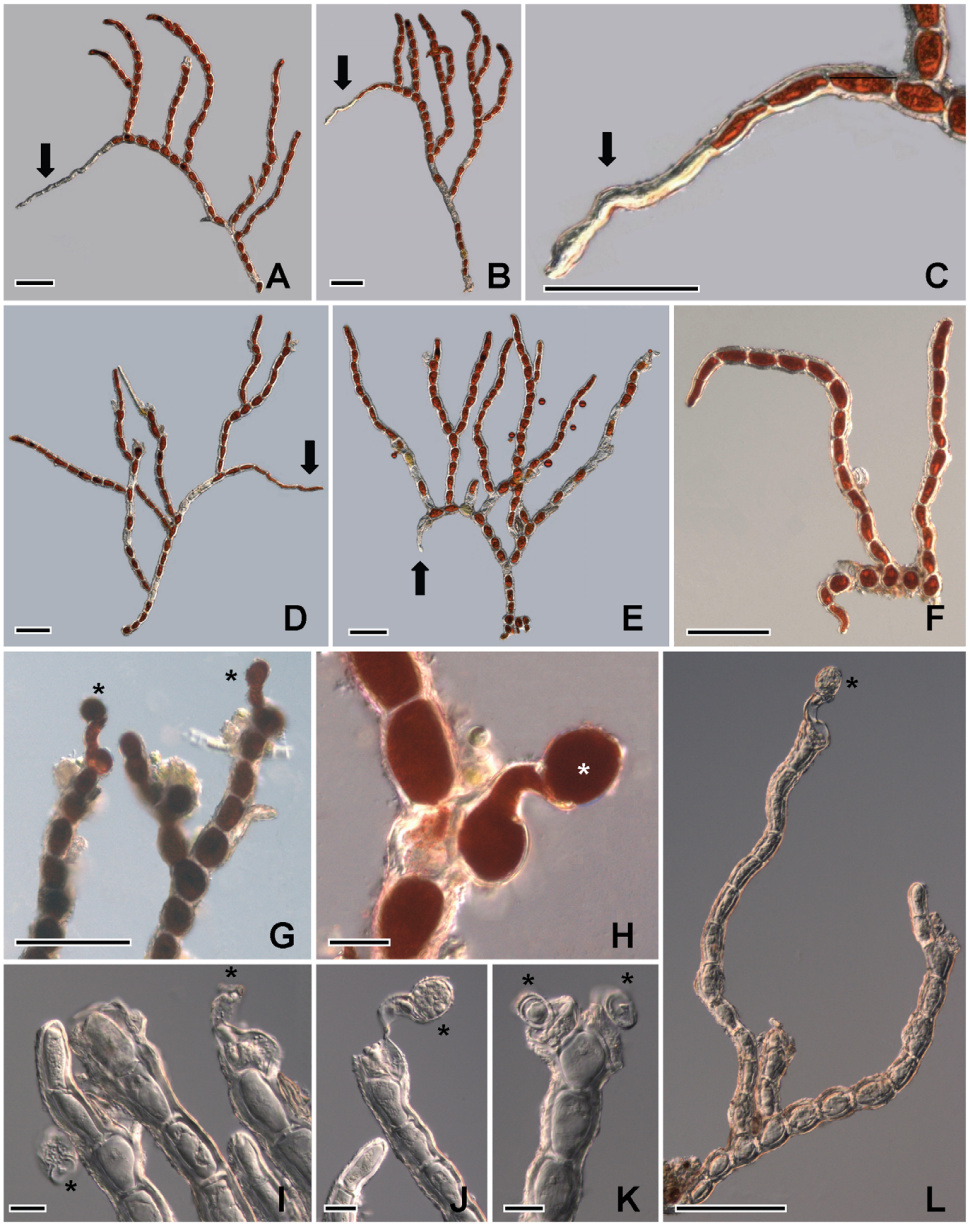
The filamentous of *Trentepohlia jolithus* var. *yajiagengensis* var. nov. 4A-4E: Filament with rhizoid (arrow); **4F:** Part of the thallus with prostrate and erect filaments; **4G-4L:** Filaments with sporangia (asterisk). Scale bars in Figs. 4G-4K are 20 µm, others are 100 µm.

**Figure 5:**
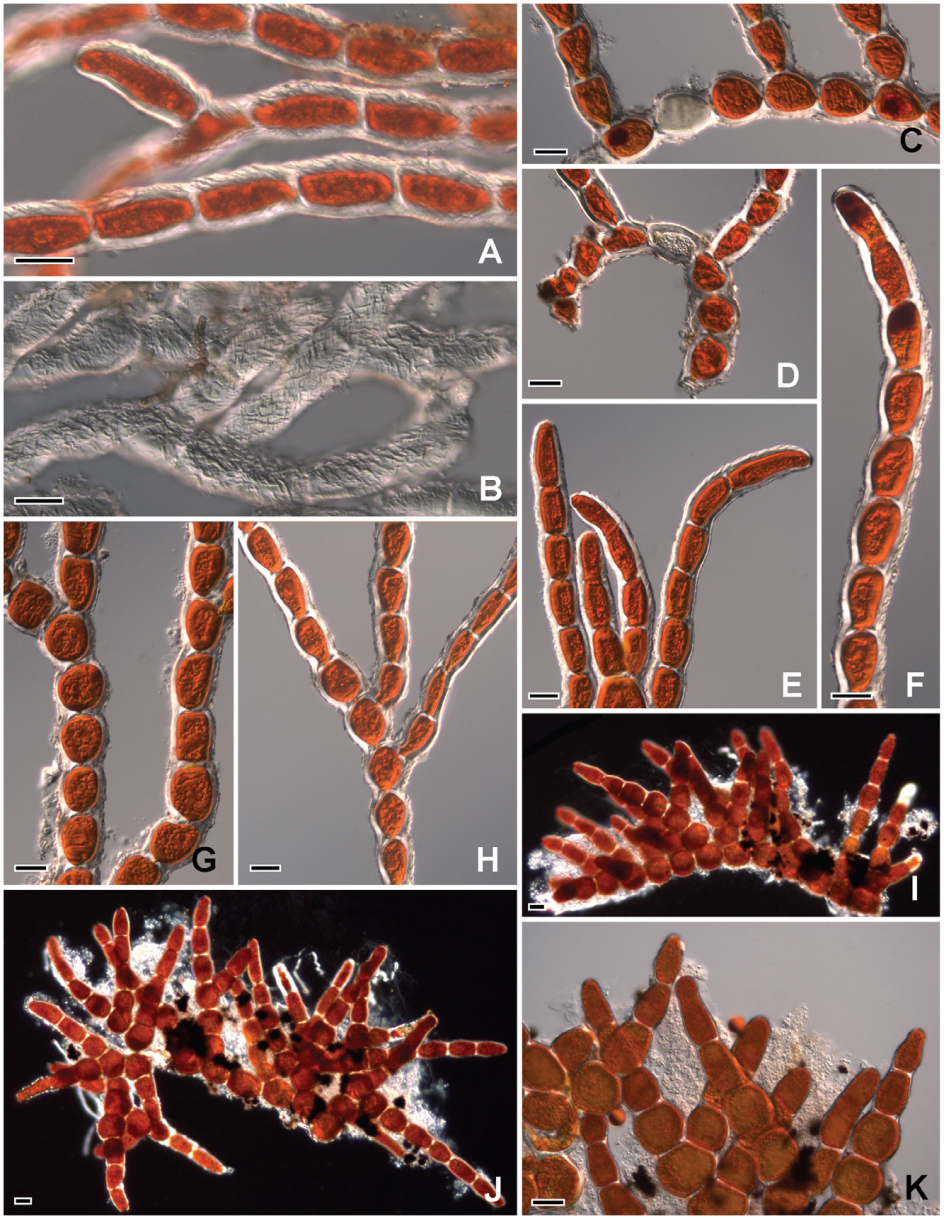
The filamentous of *Trentepohlia jolithus* var. *yajiagengensis* var. nov. 5A-5B: Coarse cell wall, the specimen of Fig. 5B was fixed with formalin and the color was bled; **5C-5D:** Prostrate cell shape; **5E-5F:** Cell shape of the upper part of erect filaments; **5G-5H:** Cell shape of the middle part of erect filaments; **5I-5K:** Culture filaments. All scale bars  = 20 µm.

The thallus forms a bright red to deep red velvety covering on exposed rocks (Fig. 2); the thallus consists of limited prostrate filaments on which well-developed erect axes are borne (Figs. 3D-3E). The prostrate part is scanty, pseudoparenchymatous, and cells subglobose or irregular in shape (Fig. 3D, Fig. 4F). In the first stage when the thallus colonized the surface of primitive rocks, the nascent filaments were all creeping, and the erect filaments originated from these prostrate filaments (Fig. 3C). The unilateral branched erect parts tended to become deeply entangled (Fig. 3B, 3D), cells more or less cylindrical (Figs. 5E-5F), often somewhat swollen in the middle (Figs. 5G-5H), 19–28×30–40 µm; branch-tips not tapering, apical cell blunt without any caps (Fig. 5E). The end cells of the main filament sometimes can form rhizoid, and the rhizoid can penetrate into the rocks (Figs. 4A-4E).The cell wall is thick, roughened with tiny reticulations (Figs. 5A-5B). In culture, the cells of the main filaments or the lower part of the branch are bigger than those in the field, the widths of these cells are up to 38 µm; the shape is close to globular. Moreover, the cell wall in culture becomes thin and smooth (Fig. 5K).

The sporangiate occurred either at the top of the axes (Fig. 4G) or lateral (Fig. 4H), single or rarely in twos (Figs. 4I-4L), globular sporangium, 21–28 µm in diameter, 24–30 µm wide.

This variety differs from the type in that its apical cells of the main filament are often faded, and become rhizoids with inversion of polarity. *T. jolithus* var. *crassior* Nordst. differs from this new variety in having much larger cell dimensions, and var. *betulina* (Rabenh.) Hariot, which grows on birch trees, in having ellipsoidal, thick-walled cells. Another variety var. *bovina* (Flot.) Rabenh. is considered by Printz 1939 to be merely a young form of the species, longitudinal thickenings, bent stalk of the hakensporangia, which are usually lateral, and the presence of caps. In var. *anthonyi* Sarma 1986, the hakensporangia are usually lateral with the presence of caps on its apical cells.

### Phylogenetic Results

**Figure 6:**
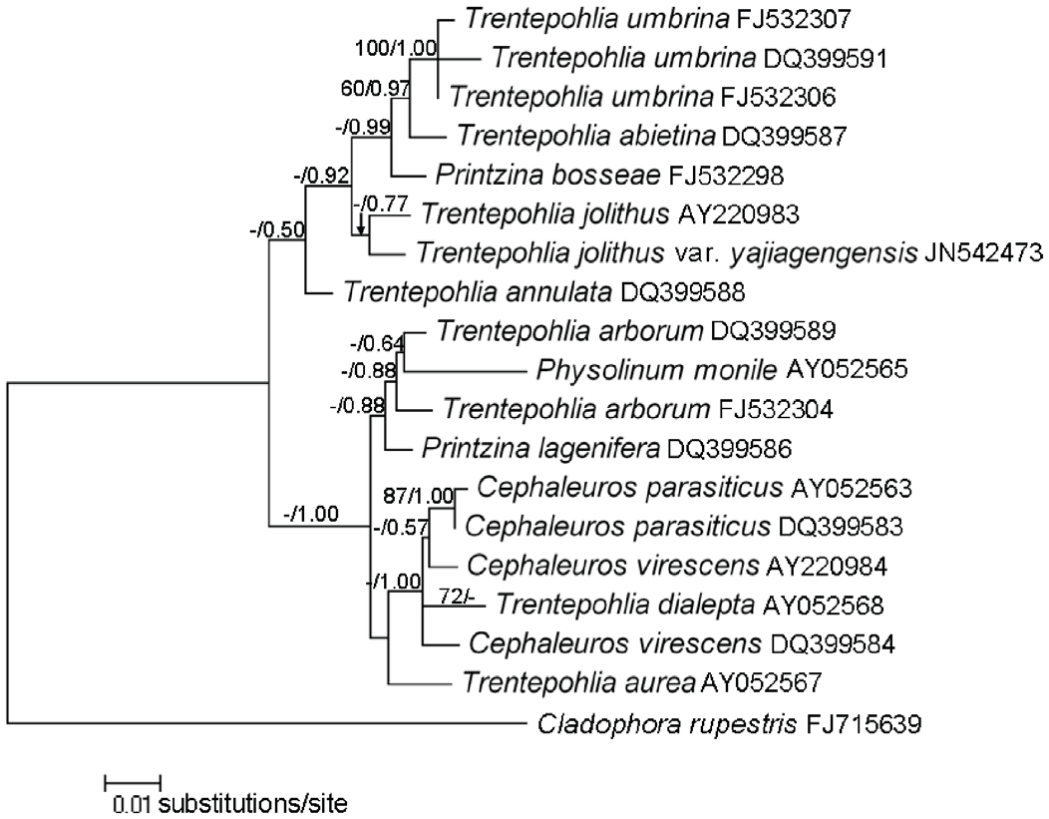
Maximum likelihood phylogenies constructed from dinoflagellates SSU rDNA sequences for trentepohlialean taxa. Numbers at nodes represent posterior probabilities/bootstrap support values from Bayesian Inference and Maximum Likelihood. Only values above 0.50 are shown.

In order to elucidate the phylogenetic position of *Trentepohlia* in nuclear-encoded SSU rDNA phylogeny, phylogenetic analysis was performed with a dataset of 19 taxa and 1513 aligned characters. *Cladophora rupestris* was included as the out-group. The other taxa belonged to the order Trentepohliales. The two analytical methods yielded more or less the same topology, but only the ML tree was presented (Fig. 6). Usually posterior probability values were higher than the bootstrap values.

The partial SSU rDNA sequence from *T*. *jolithus* var. *yajiagengensis* (JN542473) showed 98.6% sequence similarity with a sequence from *T*. *jolithus* (AY220983).

The SSU phylogeny suggested that *Trentepohlia* morphospecies located in four different clades. A large clade which included morphospecies *Trentepohlia umbrina*, *T*. *abietina*, *T*. *annulata*, *T. jolithus* and *T*. *jolithus* var. *yajiagengensis* clustered with *Printzina bosseae*. In the second clade, *T. arborum* clustered with *Printzina lagenifera* and *Physolinum monile*. Two solitary sequences associated to the morphospecies of *T*. *dialepta* and *T*. *aurea* formed separate clades with low support, respectively.

## Discussion

### Phylogenetic Position of the New Variety

SSU phylogeny indicated that the genus *Trentepohlia* was polyphyletic. In both maximum-likelihood and Bayesian analyses, *T.* *jolithus* clustered with the variety *T*. *jolithus* var. *yajiagengensis*, although the clade was poorly supported. The type species *T*. *aurea*, which should be considered the authentic genus, seemed to be distinct from other *Trentepohlia* species and form a separate branch. The members of *Trentepohlia* are phylogenetically closer to members of *Phycopeltis* and *Printzina* than to *T*. *aurea*. This suggests that a major rearrangement at the genus level might be necessary in the future. The type of substratum colonized is considered as an important taxonomic criterion for *Trentepohlia*, but species growing on different substrata did not form separate clades (e.g. epilithic *T.* *jolithus* and the new variety, corticolous *T.* *abietina*). *Cephaleuros* was paraphyletic in SSU rDNA phylogeny because *T*. *dialepta* clustered with *Cephaleuros* species; however, some authors have suggested that *T*. *dialepta* should be transferred to *Cephaleuros* [18].

### Distribution and Ecology

Basically *Trentepohlia* is distributed in tropical and subtropical areas [19]. However, *T. jolithus* is also common in temperate regions [8]. The species is the most common representative of the genus in western Ireland, which is a cold-temperate area characterized by high levels of rainfall and humidity. This area has a very mild climate in comparison with other areas of northern Europe, with temperatures ranging between 0 and 20°C, relatively limited seasonal variation, and generally lack of snow and ice [6]. *T. jolithus* var. *yajiagengensis* seems perfectly adapted to the environmental conditions of Mt. Gongga. The variety is only found on the rock surface at alt. 1900–3800 m. According to the weather station at 3000 m where climatic conditions resemble the collection areas of theYajiageng valley, the mean monthly temperature ranges from −4.5°C in January to 12.7°C in July [16]. In summer, the glaciers of the northern slopes of Mt. Gongga bring to the valleys a wet and foggy air environment and the annual average relative humidity is above 90% [14] which is ideal for *Trentepohlia* growth. This variety can even survive on rocks covered by ice in winter. In addition, we speculate that the distribution of the new variety is likely to be more extensive than the survey scope: the variety may well be found in other locations on Mt. Gongga with a similar habitat. In fact, many pictures on the Internet taken by tourists show that there are some other small-scale Red-Stone-Valleys also to be found in similar habitats to the Mt. Gongga area.

In New Zealand, *T*. *jolithus* grew on dead wood such as wooden fencing poles or power poles, failed to grow on any living plant, and very rarely grew on bare rocks [8]. In western Ireland, the species of *Trentepohlia* differed in their preference for particular substrata, usually forming well-developed populations only on a particular surface type. This species grew most commonly on old concrete or cement walls, with a very wide intervening space [6]. In this investigation, *T*. *jolithus* var. *yajiagengensis* grew most commonly on native rocks. It never grew on plants and dirty rocks or concrete walls. Figure 2D, shows that *Trentepohlia* was not found on the concrete part of the road embankment, whilst it bloomed on nearby rocks.

The production and accumulation of a high content of carotenoids is commonly found in the cells of *Trentepohlia* species [20], [21]. The spectacular bloom, due to *T*. *jolithus* var. *yajiagengensis*, has attracted many tourists to the area for the brightness and intensity of the red colour. The red colouring was caused by accumulated carotenoids (maybe astaxanthin, but we did not analyze this in detail, the absorption peak of the whole extract spectrum is 480 nm, data not shown). These carotenoids helped the variety to have resistance to strong UV radiation in the high-altitude mountain region. The variety was also rich in oils (Fig. 4E) [22], which could help the cells in being highly resistant to cold dry winter.

### The Formation of Red-Stone-Valley and the Future of the *Trentepohlia* Bloom

The formation of the massive biomass of *Trentepohlia* currently observed in the glaciated valley is related to climate change, especially global warming, glacier retreat and heavy rain. Small-scale *Trentepohlia* populations had occurred in the Yajiageng valley before 2005. In 2005, there was a serious debris flow in the Yajiageng river caused by glacier melt and rain [23]. The debris flow brought with it numerous fresh boulders, and exposed and cleaned the surface of existing rocks. *T*. *jolithus* var. *yajiagengensis* became the most abundant pioneer species on the bare rocks. Owing to adaptations to environmental factors such as the cold and, strong UV radiation, the variety grew rapidly and has formed large-scale blooms in the valley. In fact, in winter other species of *Trentepohlia* also have a high content of carotenoid, the lower temperatures and cloudless clear sky in winter provide perfect conditions favoring the growth of *Trentepohlia* [24]. Under the current environment, the pioneer community predominantly composed of *T*. *jolithus* var. *yajiagengensis* would give way to a climax community during the succession process. However, debris flows, which may reoccurr periodically in the valley, might disturb the habitat and interrupt this succession process. This variety would undergo the same succession process yet again afterwards.

## Materials and Methods

### Ethics Statement

No specific permits were required for the described field studies: a) no specific permissions were required for these locations/activities; b) locations are not privately-owned or protected; c) the field studies did not involve endangered or protected species.

### Field Studies, Cultures and Light Microscopy

On October 24, 2010, samples of *Trentepohlia* were collected in the Yajiageng valley, Mt. Gongga, Sichuan Province, China (Fig. 1, map from Google™ earth [25]), where the red *Trentepohlia* bloom could be easily recognized by the naked eye. Samples were collected by removing the thallus from substratum with a sharp knife. The materials were kept dry in centrifugal tubes using silica gel, until examination in the laboratory. Moreover, several small stones covered by the red algae were sealed in plastic collection bags. When conserved in this way, trentepohliacean algae usually remain viable for several weeks.

Voucher specimens have been deposited in the Freshwater Algal Herbarium (HBI), Institute of Hydrobiology, Chinese Academy of Sciences, Wuhan, China.

An attempt to isolate unialgal cultures was made by excision of vegetative tips and cultivating it on solid BG11 plates [26]. The plates were kept at 18°C, 14∶10 h light:darkIsolation into unialgal cultures was attempted by excision of vegetative tips. The isolate was cultivated in a Petri dish, which was placed at 18°C, 14∶10 h light:dark, 80 µmol photons m^−2^ s^−1^.

Micrographs were taken with a Zeiss Stemi 2000-C stereomicroscope equipped with Zeiss AxioCam MRc digital camera and a Leica DM5000B microscope equipped with Leica DFC 320 digital camera.

### Nomenclature

The electronic version of this article in Portable Document Format (PDF) in a work with an ISSN or ISBN will represent a published work according to the International Code of Nomenclature for algae, fungi, and plants, and hence the new names contained in the electronic publication of a PLoS ONE article are effectively published under that Code from the electronic edition alone, so there is no longer any need to provide printed copies.

The online version of this work is archived and available from the following digital repositories: PubMedCentral (www.pubmedcentral. nih.gov/), LOCKSS, and Solanaceae Source: a web resource for the nightshade family (http://www.solanaceaesource.org).

### DNA Extraction, PCR Amplification and Sequencing

DNA was extracted from trentepohliacean samples which were kept in centrifugal tubes. DNA extraction protocols were the same as those used in Mei et al. (2007) [28]. PCR amplifications were done using 5 µL of template DNA, 1×PCR buffer, 0.2 mM of dNTP, 0.4 µM of each primer, and 1.25 U of Taq DNA Polymerase (ExTaq, TaKaRa) in 50 µL total volume reactions. PCR amplification and sequencing of 18S rDNA was performed using primers designed for both algae and plants [29]. The SSU PCR started with 5 min at 94°C, followed by 35 cycles of 1 min at 94°C, 1 min at 56°C, 2 min at 72°C, ending with a final hold of 7 min at 72°C. PCR amplicon was cleaned using E.Z.N.A.™ Gel Extraction Kit (Omega, USA). The PCR product was run on an ABI 3700 sequencer (Applied Biosystems, USA). The sequence was deposited with GenBank under the Accession no. JN542473.

### Sequence Alignment and Phylogenetic Analyses

After the elimination of identical and apparently erroneous sequences, we created a set of alignments by Clustal X (v1.8) [30] and Bioedit (v7.0.9.1) [31]. Phylogenies were estimated using Maximum Likelihood (ML) and Bayesian Inference (BI) as implemented in Paup 4.0* (v4.0b10) [32] and MrBayes (v3.1.2) [33], respectively. The program Modeltest (v3. 7) [34] was used to explore the model of sequence evolution that best fits the data set by the hierarchical likelihood ratio test (hLRT) [35]. The model selected was TrN+I+G. ML analyses were performed with the heuristic search option with random addition of sequences (100 replicates) and a branch-swapping algorithm (tree bisection-reconnection). All Bayesian Markov Chain Monte Carlo (MCMC) analyses were run with seven Markov chains (six heated chains, one cold) for 1,000,000 generations. Trees were sampled every 100 generations. We obtained posterior probability (PP) values for the branching patterns in BI trees as well as bootstrap (BP) values in ML trees.

## References

[1] Chapman RL (1984). An assessment of the current state of our knowledge of the Trentepohliaceae.. In: Systematics of the green algae (Ed. by D.E.G. Irvine & D.M. John), 233–250. Academic Press, London..

[2] Rindi F, Sherwood AR, Guiry MD (2005). Taxonomy and distribution of *Trentepohlia* and *Printzina* (Trentepohliales, Chlorophyta) in the Hawaiian Islands.. Phycologia 44, 270–284.

[3] Krishnamurthy V (2000). Algae of India and neighbouring countries. I. Chlorophycota. Science Publishers, Enfield, New Hampshire.. 210 pp.

[4] Printz H (1939). Vorarbeiten zu einer Monographie der Trentepohliaceen.. Nytt Mag Naturvidensk.

[5] Printz H (1964). Die Chaetophoralen der Binnengewässer: eine systematische Übersicht.. Hydrobiologia.

[6] Rindi F, Guiry MD (2002). Diversity, life history and ecology of *Trentepohlia* and *Printzina* (Trentepohliales, Chlorophyta) in urban habitats in Western Ireland.. J Phycol.

[7] Thompson RH, Wujek DE (1997). Trentepohliales: *Cephaleuros*, *Phycopeltis* and *Stomatochroon*. Morphology, Taxonomy and Ecology. Science Publishers, Enfield, New Hampshire.. 149 pp.

[8] Sarma P (1986). The freshwater Chaetophorales of New Zealand.. Beihefte zur Nova Hedwigia.

[9] Saxena PN (1961). Algae of India. 1. Chaetophorales. Bulletin of the National Botanical Garden no. 57. Lucknow, India.. 60 pp.

[10] Akiyama M (1961). Aerial and terrestrial algae in San-in Region of Honshû, Japan Bull Shimane Univ (Nat Sci).

[11] Fisher A (1922). Die *Trentepohlia*-Arten Mährens und West-Schlesiens.. Ősterr Bot Zeit.

[12] Prescott GW (1963). Algae of the Western Great Lakes area..

[13] Li Z, He Y, Yang X, Theakstone WH, Jia W (2010). Changes of the Hailuogou glacier, Mt. Gongga, China, against the background of climate change during the Holocene.. Quatern Int.

[14] Lu X, Cheng G (2009). Climate change effects on soil carbon dynamics and greenhouse gas emissions in Abies fabri forest of subalpine, southwest China.. Soil Biol Biochem.

[15] Zhong XH, Luo J, Wu N (1997). Researches of the forest ecosystems on Gongga Mountain. Chengdu: Chengdu University of Science and Technology Press.. [in Chinese with English summary].

[16] Huo C, Cheng G, Lu X, Fan J (2010). Simulating the effects of climate change on forest dynamics on Gongga Mountain, Southwest China.. J For Res.

[17] Lü Y, Wang G (2008). Response of Runoff Variation to Climate Change in Hailuogou Drainage Basin in Gongga Mount in 1990–2007.. J Glaciol Geocryol.

[18] Rindi F, Lam DW, López-Bautista JM (2009). Phylogenetic relationships and species circumscription in *Trentepohlia* and *Printzina* (Trentepohliales, Chlorophyta).. Mol Phylogenet Evol 52, 329–339.

[19] Bourrelly P (1966). Les algues d’eau douce. Initiation á la systématique. *I. Les algues verts.* Boubee, Paris.. 511 pp.

[20] Abe K, Takahashi E, Hirano M (1998). Characteristics of growth and carotenoid accumulation of the aerial microalga *Trentepohlia aurea* in liquid culture.. J Mar Biotechnol.

[21] Kjosen H, Arpin N, Liaaen-Jensen S (1972). The carotenoids of *Trentepohlia iolithus*. Isolation of β, β-carotene-2-ol, β, ε-carotene-2-ol and β, β-carotene-2, 2′-diol.. Acta Chem Scand.

[22] Gildemeister E (1916). The Volatile Oils Vol 2.. John Wiley and Sons: New York.

[23] Chen XQ, Cui P, Chen BR, Qi YL (2006). “050811” large scale debris flow in Hailuo Valley and prevement countermeasures. Bull Soil Water Conserv 26: 122–126.. [in Chinese with English summary].

[24] Mukherjee R, Borah SP, Goswami BC (2010). Biochemical characterization of carotenoids in two species of *Trentepohlia* (Trentepohliales, Chlorophyta).. J Appl Phycol.

[25] Google Inc (2011). Google Earth, version 6.0.. Accessed 7 Sep 2011.

[26] Allen MM (1968). Simple conditions for growth of unicellular blue-green algae on plates.. J Phycol 4, 1–4.

[27] McNeill J, Barrie FR, Burdet HM, Demoulin V, Hawksworth DL (2006). International Code of Botanical Nomenclature (Vienna Code). Regnum Vegetable 146. A.R.G. Gantner Verlag KG, Liechtenstein.. Electronic version at.

[28] Mei H, Luo W, Liu GX, Hu ZY (2007). Phylogeny of Oedogoniales (Chlorophyceae, Chlorophyta) inferred from 18S rDNA sequences with emphasis on the relationships in the genus *Oedogonium* based on ITS-2 sequences.. Pl Syst Evol.

[29] Hamby RK, Sims L, Issel L, Zimmer E (1988). Direct ribosomal RNA sequencing: optimization of extraction and sequencing methods for work with higher plants.. Plant Mol Biol Rep.

[30] Thompson JD, Gibson TJ, Plewniak F (1997). The Clustal X windows interface: flexible strategies for multiple sequence alignment aided by quality analysis tools.. Nucleic Acids Res.

[31] Hall TA (1999). BioEdit: a user-friendly biological sequence alignment editor and analysis program for Windows 95/98/NT.. Nucleic Acids Symp Ser.

[32] Swofford DL (2002). PAUP*..

[33] Huelsenbeck JP, Ronquist F (2001). MRBAYES: Bayesian inference of phylogenetic trees.. Bioinformatics.

[34] Posada D, Crandall KA (1998). MODELTEST: testing the model of DNA substitution.. Bioinformatics.

[35] Huelsenbeck JP, Crandall KA (1997). Phylogeny estimation and hypothesis testing using maximum likelihood.. Annu Revi Ecol and Systemat.

